# Ethorobotic rats for rodent behavioral research: design considerations

**DOI:** 10.3389/fnbeh.2023.1281494

**Published:** 2023-12-21

**Authors:** Robert Siddall

**Affiliations:** School of Mechanical Engineering Sciences, University of Surrey, Guildford, United Kingdom

**Keywords:** ethorobotics, laboratory rodents, biorobotics, biohybrid interaction, robotic rats, ethology, bioinspired robotics

## Abstract

The development of robots as tools for biological research, sometimes termed “biorobotics”, has grown rapidly in recent years, fueled by the proliferation of miniaturized computation and advanced manufacturing techniques. Much of this work is focused on the use of robots as biomechanical models for natural systems. But, increasingly, biomimetic robots are being employed to interact directly with animals, as component parts of ethology studies in the field and behavioral neuroscience studies in the laboratory. While it has been possible to mechanize and automate animal behavior experiments for decades, only recently has there been the prospect of creating at-scale robotic animals containing the sensing, autonomy and actuation necessary for complex, life-like interaction. This not only opens up new avenues of enquiry, but also provides important ways to improve animal welfare, both by reducing or replacing the use of animal subjects, and by minimizing animal distress (if robots are used judiciously). This article will discuss the current state of the art in robotic lab rats, providing perspective on where research could be directed to enable the safe and effective use of biorobotic animals.

## 1 Introduction

**Figure 1:**
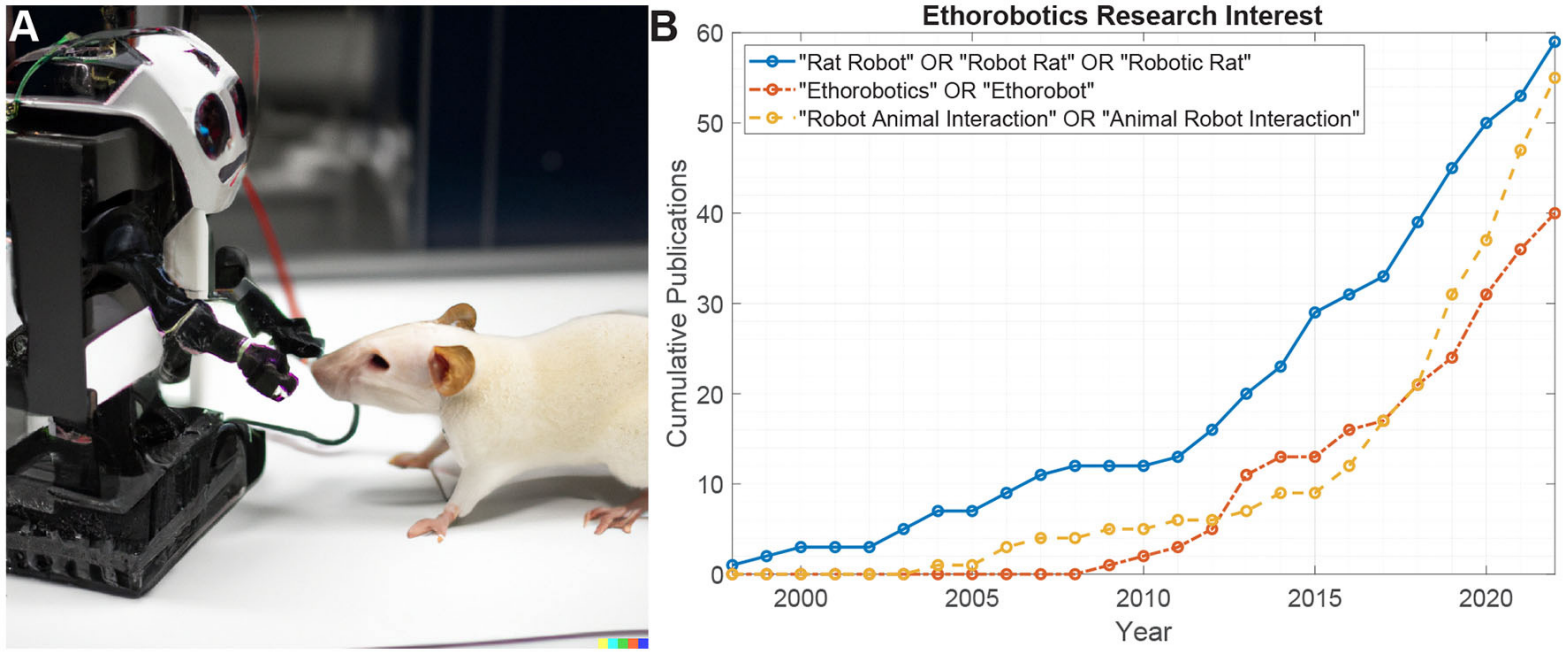
**(A)** AI-generated concept sketch of a laboratory rat interacting with a robot. **(B)** Web of science citation analysis for different search terms related to robotic rodents and ethorobotics. Robotic rats are some of the longest pursued “ethorobots”, and the most widely studied in animal-robot interaction research.

Rodents (*Rodentia*) are remarkable animals, which have successfully occupied almost every habitat on earth, and account for almost 50% of all observed mammal species. Among the rodents, the *Muridae* family are ubiquitous in the modern world, due to their commensal relationship with humans. Domesticated rats (*Rattus norvegicus forma domestica*) and mice (*Mus musculus domesticus*) have become some of the most important model animals in modern biology. In the US, as many as 100 million rodents are used in experiments in a given year (Carbone, 2021). Many of these experiments involve social behavior tests featuring multiple rats interacting with one another, which can be used (for example) to gauge the psychoactive effects of pharmaceuticals, or to phenotype animal models of neurological disorders. Unfortunately, some of these social tests face ethical issues deriving from the risk of physical harm to the animal subjects. These issues are particularly relevant in the tests featuring encounters among unfamiliar individuals. For instance, one of the most common tests of aggressivity is the resident-intruder test (Koolhaas et al., 2013; Ruzza et al., 2015), in which a stimulus rat or mouse (the intruder) is placed in the home-cage of another rat or mouse (the resident). Since rats and mice are highly territorial, the intrusion of a new rat or mouse elicits aggressive reactions aimed at territorial defense. While the variable of interest is the resident animal's reaction to the intrusion, the following fight may lead to injuries and even death of the experimental animals, raising important issues regarding animal welfare. The recent d'Isa-Gerlai rating scale for the impact on behavioral tests on animal welfare, which features 12 levels from A (animal-friendly) to L (lethal), rates the resident-intruder test K due to the risks of physical harm (d'Isa and Gerlai, 2023). The employment of robotic rodents as intruders could completely eliminate injuries deriving from fight, representing a notable refinement of aggression tests in behavioral neuroscience. Moreover, social experiments with animals are complex and face ethical issues with reproducibility. If sufficiently “life-like” robotic rodents can be developed, an opportunity is presented to simultaneously improve the repeatability with which social interaction can be tested (since the behaviors of the robotic intruder are programmable by the experimenters) and animal welfare, by reducing both the number of rodents required for a given experimental cohort and the incidence of injury to experimental subjects from violent interaction with conspecifics. But before such robot-rat interactions (Figure 1A) can enter into wide use, significant challenges remain in replicating the suite of behaviors necessary for a scientifically useful interaction.

Biorobotics is a subdiscipline of robotics which seeks to which develops biomimetic or bio-inspired robots, which may be used not only as technological applications to help society and/or the environment, but also as scientific tools for biological research, for instance through the use of synthetic abstractions of biological systems (Tamborini and Datteri, 2022). Fueled by the miniaturization of computation and sensing, and the proliferation of advanced manufacturing, the increasing sophistication with which robots can be built offers ways to test biological hypotheses in a highly controlled fashion, while also reducing the need for intrusive animal experimentation. These bio-inspired robots can be used to elucidate biomechanical principles in support of animal studies (Siddall et al., 2021), or simply to provide an accessible way of engaging a wider audience in educational projects (Siddall et al., 2023). Indeed, the growing autonomy with which robots can be imbued now offers a chance to use biorobotics in more sophisticated ways, and to test interactive effects.

The development of robots capable of interacting with live animals for behavioral studies (often called “ethorobotics") has been employed in a number of ways (Romano et al., 2018), most often through animatronic replication of body language and visual navigation cues. This is a fast growing area of research with a 5-fold growth in the past decade (Figure 1B). Rodents have a long history as model animals, and consequently robotic rats have the most research attention in robot-animal interaction studies. However, the range of animal robot interactions is rapidly growing, and recent studies have shown the influencing of collective behavior with robotic cues, e.g., in schooling fish or swarming bees (Romano et al., 2018).

Ethorobotics may provide useful tools for behavioral research. Indeed, automated mechanical systems have long been used in rodent behavioral experiments, and mobile robots have been used to manipulate the social behavior of a variety of animals, including insects (Griparić et al., 2017), fish (Polverino et al., 2013), and birds (Gribovskiy et al., 2015), not to mention the vast amount of research devoted to human-robot interaction. But it has only recently become possible to endow robots with more complex interactive behavior. Recent work has closed the loop between animal and robot in zebrafish (Bonnet et al., 2018; Khalil et al., 2019) and the techniques for animal-interactive robot control are developing rapidly (Landgraf et al., 2021), though the field is still nascent. Collectively, the work on ethorobotics to date has shown the ways in which robots can lead to more sophisticated and controllable animal experiments, and there is a clear benefit to directing more robotics work toward rodents, as they are the most widely studied animal models.

While both rats and mice are widely used as test subjects in behavioral biology, this paper will focus primarily on rats, for four principal reasons. Firstly, rats are larger than mice (200–400 g vs. 20–30 g), a size difference which is enough to make robotic rat development possible with off the shelf electronics and actuation, whereas the miniaturization needed for mouse-scale robots would currently require a drastically greater development effort. Secondly, rats are employed both as subject animals in rat studies and as stimulus animals in mouse studies, for instance in the predator threat test for mice (Blanchard et al., 1998, 2003). Thirdly, rats are more expensive to breed and maintain than mice, so their substitution would have a greater economical impact on laboratories. Lastly, mouse and rat colonies must be kept separated. The availability of robotic rats would allow laboratories endowed only of a mouse facility to perform mouse-rat interaction experiments without opening a rat facility.

## 2 State of the art in biohybrid rodent studies

**Figure 2:**
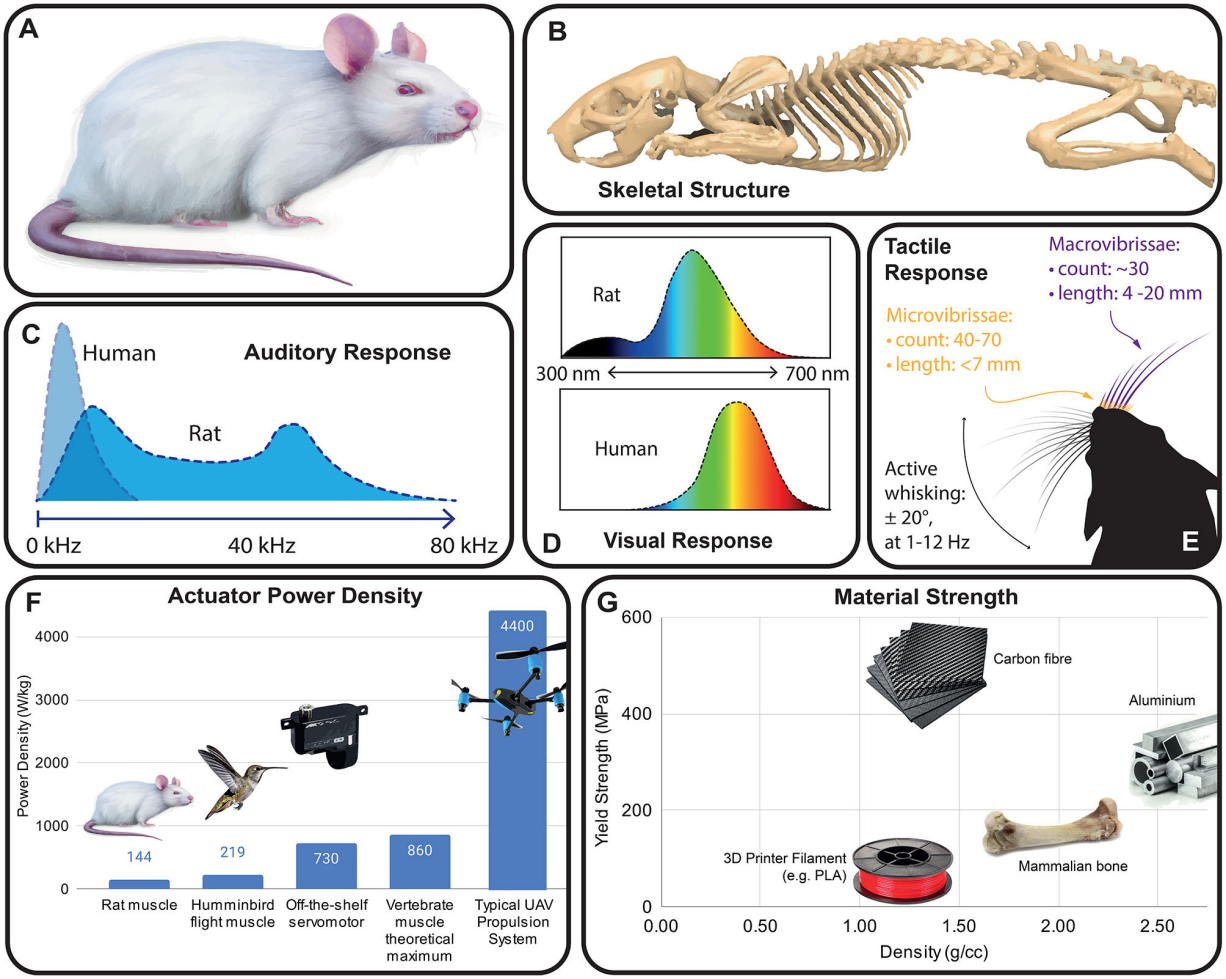
Rat anatomy and salient sensory features. **(A)** Sketch of a widely used albino rat strain (e.g., Wistar, Sprague Dawley). **(B)** Rat skeletal anatomy [CT scan data from Doney et al. (2013)]. **(C)** Audio response of a typical rat compared to human hearing [data from Kelly and Masterton (2005)]. **(D)** Normalized spectral sensitivity of rats and humans, showing effect of rat UV opsins [data from De Farias Rocha et al. (2016)]. **(E)** Vibrissae overview [data from Brecht et al. (1997)], showing macro and micro-vibrissae, and active whisking kinematics. **(F)** Mass-specific power of animal muscles vs. off the shelf electric motors [muscle data taken from Pennycuick and Rezende (1984), Martin et al. (2000), Reiser et al. (2013)]. **(G)** Typical strength vs. density for common robotics materials vs. animal bone.

To date, around a dozen different prototype rat robots have been presented in the literature, many of which have been tested interacting with live rats in multiple follow-up studies. Table 1 collects body dimensions, movement speeds and estimated power consumption (based on battery provision) for various robotic rodents. Table 1 is limited to rodent-mimicking hardware designed with animal interaction in mind—several other works exist exploring rodent interaction with off the shelf robots (e.g., Del Angel Ortiz et al., 2016), using rat-biomimicry to develop novel hardware (e.g., Pearson et al., 2007), implementing rat-like behaviors to test neuromechanical hypotheses *in-silico* (e.g., Fend et al., 2004), or testing virtual robots (Merel et al., 2019). While many robots take the approach of attempting to replicate as directly as possible the kinematics of rat movement, currently the robots most widely tested in animal interactions use simplified geometry and rely on wheels (Shi et al., 2011, 2012, 2015; Wiles et al., 2012; Heath et al., 2018; Yamada et al., 2021). This allows them to move at similar speeds to rats over engineered/flat surfaces (~1 m/s), but comes with the cost of being unable to move over more complicated terrain, or to replicate rat body postures (e.g., rearing Yuanzhong et al., 2022). Most of the robots in Table 1 use off the shelf servomotors which are liable to produce ultrasonic noise, and of the robots in Table 1, only “PiRat” has been specifically designed to minimize motor whine in rat auditory frequencies. Quadrupedal robots (Laschi et al., 2006; Ishii et al., 2009a,b; Lucas et al., 2019; Shi et al., 2022) thus far have not been able to attain biological movement speeds. Rats move with a lower center of gravity, with more bent limbs (Figure 2B) and different hindlimb kinematics compared to the cursorial animals widely studied for quadrupedal locomotion (e.g., dogs), and more research is needed to adapt the compliance and force control needed for efficient legged locomotion. Soft robotics techniques are present in existing rat robots (Lucas et al., 2019; Shi et al., 2022; Yuanzhong et al., 2022), but of the legged robots in Table 1, only one (Lucas et al., 2019) has a design giving close attention to limb compliance. Given the vast amount of data and analysis of rat running, and the availability of simulated neuromechanical models (Merel et al., 2019), there is ample material available for further robotic development.

**Table 1:** Comparison of the designs of previously developed robotic rats, with a column indicating which robots have been tested interacting with a live rodent.

**Robot**	**Mass**	**Speed**	**Size**	**DOF**	**Locomotion**	**Power**	**Interaction**	**References**
	**(*g*)**	**(*m/s*)**	**(*mm*)**		**type?**	***(W)***	**tested?**	
Psikharpax	–	0.3	500	~10	Wheels	–		Meyer et al., 2005
Rat-Robot	340	–	146	13	Legs	~10	✓	Patanè et al., 2007
WR-1	1150	0.03	270	15	Legs	–	✓	Ishii et al., 2009a
WR-2	850	0.02	240	15	Legs	16		Ishii et al., 2009b
WR-4	850	1	270	10	Wheels	–	✓	Shi et al., 2011
WR-3	1,000	1	240	14	Wheels	–	✓	Shi et al., 2012
iRat	600	0.5	180	2	Wheels	9	✓	Wiles et al., 2012
WR-5	700	1	240	13	Wheels	15	✓	Shi et al., 2015
PiRat	240	1.1	123	2	Wheels	–	✓	Heath et al., 2018
NeRmo	275	0.3	117	13	Legs	18		Lucas et al., 2019
WR-7	–	0.24	230	3	Wheels	–		Yamada et al., 2021
Soft Rat	–	–	240	6	None	–		Yuanzhong et al., 2022
SQuRo	220	0.2	136	12	Legs	12		Shi et al., 2022

Robotic legged locomotion cannot yet produce life-like movement speeds, and so many robots employ wheels.

As well as allowing more life-like and efficient motion for legged robots, the use of elastic elements limits force and offers intrinsic safety. However, social interaction does not necessarily require absolute biomechanical mimicry. The “iRat” platform (Wiles et al., 2012) has already been used in a cooperative interaction and has been proven effective in inducing a positive response in live rodents (Quinn et al., 2018), while an undisguised legged robot was used to elicit faster learning in a Skinner's box experiment (Patanè et al., 2007). Among the robots that have been presented, the only instance of a pro-social response was found in Quinn et al. (2018), when rats showed a preference for helping a trapped robot that was made to move in a social fashion over a randomly moved robot. However, it cannot be established that the robot was treated as a conspecific by the rats, or whether another motivation was present [rats actively work to access enrichments (National Research Council, 2008), for example]. More effort is needed to establish the extent to which an inanimate object can be recognized socially by a rat, and hence understand whether disguising of robots is effective/necessary.

## 3 The sensory environment of a laboratory rat

Rats navigate their environment in a way which is alien to humans (Burn, 2008), and as a consequence is not intuitive to robot designers (Figure 2). While many laboratory rats appear markedly different from their wild relatives (Figure 2A), even domesticated species have been shown to retain many of the behaviors of their “wild” suite when allowed to express them (Berdoy, 2002). Rats are nocturnal, with elongated bodies adapted for stealth and access to confined spaces (Figure 2B). They are short-sighted and possess limited color vision (Figure 2D), but have highly developed olfaction, gustation and hearing (Figure 2C) as well as a sophisticated suite of tactile senses, including vibrissal perception (Figure 2E). These senses have different sensorial ranges, and here we will go through them in approximate order of the sensorial range.

Of the suite of primary rodent senses, perhaps the most difficult to control and interpret in a laboratory environment are olfaction and gustation, which unfortunately also provide rats with some of their strongest high-level navigation cues. Rodents sniff at a frequency of up to 12 Hz (Spencer et al., 2021) (representing a significant data rate) and smell directly affects social response [notably for interaction experiments, smell affected cooperation in Gerber et al. (2020)]. The miniaturization of “e-nose” sensors, and the growing ease with which artificial intelligence can be applied to their data implies that better biomimetic smell classification in robots is well within reach of current technology. Indeed, it has been possible to classify certain rodent odors with electronic noses for decades (Montag et al., 2001). However, the ability of rodents to localize and track scents is far more complex, involving the exploitation of airflow differences between nostrils and active “casting” of the nose to trace the source of olfactory cues, and rat-like chemotaxis is currently considerably more challenging for an artificial system. Importantly, olfaction could not be required in the robotic rat if it used not as subject animal, but rather as stimulus animal for living rats. Nevertheless, emission of scent could be fundamental for appropriate social responses from living rats.

After scent or lack thereof, the immediately obvious problem with the use of an electromechanical rat is its audio signature. Rats' large hearing range (up to 100 kHz, Figure 2C) implies that the whine of electric motors is much more audible. Off the shelf servomotors typically used in robotics (and in many of the robots in Table 1) use control pulse frequencies of around 9 kHz, leading to a “whine” at the same frequency. While many more sophisticated drivers use frequencies of up to 40 kHz (making the motor whine inaudible to humans), most motor drivers would need to be operated at their maximum viable frequency to be fully inaudible to rats. Additionally, the lower frequency noise from gearboxes and other mechanisms should be considered.

Just as the differing auditory response of rats has implications for robot design, so does the broadened rat visual response, in particular the presence of UV sensitive opsins (Figure 2D). A differing spectral response means that color as perceived by humans is not a reliable basis for mimicry. Rat fur is known to fluoresce under UV light (Tumlison et al., 2021) and has even been suggested to provide rudimentary sensation of infrared radiation (Baker, 2021). Fur maintenance is clearly important to rats, who spend 50% of waking time grooming (Lambert, 2011).

Finally, rats' sense of their environment is strongly tactile. The upper lips of rats contain around 100 vibrissae/whiskers (Figure 2E), whose follicles are dense with nerve endings and which remain constantly in motion as the animal moves (Brecht et al., 1997). Whisking of rat vibrissae at up to 12 Hz gives the animals constant information about positions and textures of the substrates they move across, and their tactile response is sufficiently sensitive to accurately measure airflow (Yu et al., 2016). Interestingly, Tony Prescott's laboratory at the University of Sheffield (United Kingdom) has designed a biomimetic whisking scratchbot capable of tactile sensing through active movement of the vibrissae, on the model of the rat whisker sensory system (Pearson et al., 2007, 2010; Prescott et al., 2009; Fox et al., 2012). While providing a robotic rat with complex tactile abilities would not be necessary if the robotic rat is employed as stimulus animal, the presence of whiskers and of whisker movements could be particularly important to induce adequate social reactions in living rats.

## 4 Design considerations for robotic rats

From a coarse engineering perspective, the power provision necessary to replicate rat locomotion is within the power and energy density limits of current electrical actuators and batteries. Maximal exertion observed in respirometry tests of running rats is roughly 3 kcal over 30 min, or 7 W of average output power (Paes et al., 2016). This could be delivered with 2A of current from a single lithium battery, with a 50 gram battery being sufficient for an hour of high-intensity exercise in a hypothetical rat-like robot. This is significantly less power than is typically used in robotic rats (mean robot power in Table 1 = 13.4 W) despite their lower movement speeds, highlighting the gap in locomotion efficiency that currently exists between animal and robot. Yet, mammalian muscle power output reaches around 200 Watts per kilogram muscle mass, which is below the peak performance of high-end hobbyist servo motors (~700 W/kg), and well below high performance brushless motors (Figure 2A). Similarly, engineered materials can readily match or exceed the specific strength of animal bone (Figure 2B), although it should be noted that bone has a flexibility which can only be matched by composites maintaining a strength similar to animal bone.

This is not to trivialize the challenge of replicating rat locomotion; power provision is not the metric for success in biohybrid robot design—e.g., the compliance, distributed sensing and evolutionary tuning of muscoskeletal systems have profound effects on locomotion ability (Spröwitz et al., 2013). This simply means that many observed behaviors in rats are potentially mechanically replicable without requiring novel miniaturization of available technology. Size is important; larger rats will attempt to dominate smaller rats in social encounters, and so social interaction requires that robots be miniaturiseable to a sufficient extent that they do not intimidate (without sacrificing mobility) and it is encouraging that current actuation is sufficient to achieve this goal.

To date, a strong focus of rat robot design has been on kinematic similarity to the animal. This is useful to replicate natural movement and interaction behaviors—rat interaction is often physical, and has distinct “choreographies” (Lambert, 2011) that life-like interactors will need. However, far less attention has been given to assimilating robots to the full suite of rodent senses, in order to enhance the probability that they will induce the intended behaviors in living rats. Humans have a profound visual bias, and more attention could be given to mimicking other aspects of robotic rat's “appearance", including audio, scent and tactile similarity. Additionally, the robot rat could be endowed with senses itself and this could make its behavior even more natural. For instance, the first phase of most social interactions involves exploration of the perimeter of the interaction space by both the resident and the newcomer rat. Fortunately, basic wall-following is one of the easier behaviors to replicate in a robot with contact switches or proximity sensors (this a common feature of robotic vacuum cleaners, for example).

One advantage that any rat robot has is the ability to use external sensors to augment its perception. Automated behavior tracking has been in use in laboratory ethology for over 20 years (Isik and Unal, 2023), and has rapidly improved with the advent of deep-learning (Mathis et al., 2018; Nilsson et al., 2020). External gas sensors, microphones and cameras can all be used to choreograph robot behavior within an enclosure and augment computational power, so there is limited need to compact processing power into a mobile robot beyond convenience/transport. In addition, many behaviors could be remotely-controlled directly by the experimenters, which could decide in real time the most appropriate reaction for the robotic rat. Consequently, rather than providing the rat robot with a complex embedded sensorial system, the primary considerations should be locomotion and appearance.

Regarding movement, rodents commonly live in cages endowed with a bedding of soft sawdust. Existing robotic rats typically can only move on smooth, flat surfaces and are incapable of locomotion on sawdust. While some social tests can be performed outside the home-cage, other tests (such as the resident-intruder test) must be performed in the home-cage of the resident animal. This issue could be easily solved by placing a rubber mat or a transparent plexiglass sheet on the sawdust of the home-cage before the test, which would give the robotic rat the possibility to walk.

Rat robots may also need to reflect rat behavior in order to provoke a natural response. The series of rat robots developed at Waseda University (Table 1) devote considerable design effort to accurately mimick the posture and rearing behaviors of natural rats, and elicited statistically significant response behaviors in live rats. However, many rat posture cues are much more subtle than rearing: ear wiggling, whisker protraction, eye tightening and mouth opening are all present during social interaction (Ebbesen and Froemke, 2021). Unlike the primary muscles used for locomotion, replicating facial muscles weighing fractions of a gram represents a technological challenge. Mimicking facial expressions at scale would likely require the use of novel actuation (e.g., shape memory alloys or other smart actuators).

Outside of technical considerations, several features of modern robotics research practice could be ethically problematic when transposed into biological research. Firstly, it is not typical for robotics research articles to require full reproducibility of the prototypes of the authors. Full sharing of the code, design files, and manufacturing instructions needed to reproduce robotics work is uncommon in published articles - the reporting standard is the minimum technical detail needed for comprehension, not for full replication. Secondly, design for decontamination is not typically required of research robots, and widespread use of robots in rodent studies will either require design for disposability or levels of ingress protection normally reserved for medical robots, which would allow the use of detergents and alcoholic solutions to clean and disinfect the rat robots.

Finally, the prototyping-led, Edisonian design approaches common in robotics research do not lend themselves to judicious use of animal subjects, nor the minimum level of scientific quality needed to justify the use of living creatures. Establishing new robotic experiment paradigms will require extensive animal testing with a sufficient number of subjects. Existing ethical guidelines establish general best practice, but have little to no information on the use of animatronics (Van Sluyters and Obernier, 2003). However, if animatronic robots will be used to interact with living animals, important safety criteria need to be considered and should become part of the robot design requirements. For example, since rodents often explore new items by mouthing them, it is important that no elements on the surface of the robot can be bitten off or ingested by the animals. Indeed, robot prototypes should be used with animals only after safety considerations have been implemented in their design.

## 5 Conclusion

While there are vastly more avenues of enquiry into robot-rat interaction than can be collected into a perspective article, surveying the literature suggests some key topics that need research attention before robots can be effectively deployed as tools for behavioral research:

- The extent to which it is possible to elicit social behaviors in a rat with an inanimate object has not been established. Research into rodents and other taxa has demonstrated the importance of biomimetic appearance and movement (Landgraf et al., 2021), but controlled tests of the relative importance of appearance, sound, scent, and posture are needed to establish clearer design requirements for robots. The “helping behavior” paradigm (Bartal et al., 2011), in which a “trapped” robotic rat induces rescue behavior in a living rat, has already been employed with robots (Quinn et al., 2018) and provides a safer initial way to test rat responses without requiring direct animal-robot contact (a “trapped” robot also reduces locomotion performance requirements).
- Appropriate manipulation of olfaction is a consistent challenge in rodent studies, and is particularly acute in robot interactions. To date, robot interaction studies have used neutral scent marks to distinguish robots (Quinn et al., 2018), but given the importance of scent to social interaction, effort should be put into testing the integration of scents which have a more reactive effect on the subject animals.
- The use of compliant/soft elements in robotic rats should be increased. Soft structures improve the intrinsic safety of interacting robots, as well as providing locomotion benefits. To date only minor elastic elements have been employed in robotic rats.
- Finally, to create repeatable, reproducible experiments, robot autonomy is required. Using human operated robots will be sufficient to make progress in the areas listed above, but behaviors will ultimately need to be fully automated, so that generalizable experiments can be run by different researchers across institutions. Automated chasing of an individual by a robot is already possible (Heath et al., 2018), but a fuller suite of robot postures/responses requires subtler timing and perception, almost certainly requiring advanced machine learning for visual classification of animal behaviors, which is an already an active topic of research (Nilsson et al., 2020), but not yet integrated into robot development.

Current rodent behavioral tests of social behavior (such as interaction with unfamiliar subjects or the resident-intruder test) may lead to fight and consequently to pain and injury of the animals. The employment of robotic rats in social interaction tests, especially the tests of aggressivity (as the resident-intruder test), can avoid the risks for the health of the subjects and would notably improve animal welfare. In the resident-intruder test, if a robotic rat is used as stimulus rat (the intruder), wounds and deaths deriving from fight could be completely avoided and the aggressive responses of the subject rat (the resident) could be measured in total safety. This would be an enormous refinement of aggressivity tests in behavioral neuroscience. Moreover, several characteristics of the robot stimulus animal (such as the posture and the vocalizations) could be controlled by the experimenters, to better understand the effects of single behavioral variables on the social behavior of the subjects. In addition to improving both the ethical and experimental quality of animal testing, the fact that rats are already widely used as models for psychological disorders implies that there are new avenues of inquiry that could be opened up by more controllable interaction studies. Rats derive real and measurable health benefits from social interaction (Hermes et al., 2009), and robotic rats may provide a more controllable means of ameliorating stress in isolated captive animals. Furthermore, the insights gained in robot-rat interaction may also find application in conservation breeding programs, particularly for endangered species, with robotic predators allowing appropriate fear-conditioning of individuals before release into the wild. Indeed, technological conservation tools have recently been defined as “the next generation of engineering-biology collaborations” (Schulz et al., 2023).

In this article the many practical advantages that could be brought by hypothetical rat robots have been outlined, but the question of what type of knowledge robotic rat experiments would yield deserves further attention. The level of sophistication at which a robotic animal moves from being a particularly complex but still fundamentally mechanical experiment to being a viable simulation of animal-animal interaction is not clear at this stage. Even if a robot was shown to convincingly replicate social interaction, it would still be necessary to determine what behavioral features may have been lost because of the abstractions and simplifications inherent in any synthetic copy of a living animal.

Finally, it should also be noted that robotic rat research could lead to a cross-fertilization between biology and robotics. Biology could benefit from the applications deriving from robotics, and robotics could benefit from biological knowledge to develop bioinspired prototypes. At a basic engineering level, rats are remarkably adaptable animals, with abilities to traverse terrain that would benefit many robotics applications such as inspection and non-invasive ecological monitoring. Such a well-studied animal as the rat should be a target for biomimetic roboticists even without the significant potential direct benefits to biology research. In order to achieve such cross-fertilization, a cross talk between robotics engineers and biological scientists should be started. This could be done by increasing the participation of biological scientists in robotics congresses such as Living Machines (Hunt et al., 2022) and the long-running From Animals to Animats (Cañamero et al., 2022), and of robotics engineers in biological congresses such as the ones of the Society for Neuroscience (SfN) and of the Federation of European Neuroscience Societies (FENS). Robotics engineers should try to write in biological journals and biological scientists should try to write in robotics journals. Collaborations between robotics labs and biological labs should be promoted. Funding agencies could launch funding offers for such collaborative projects. Indeed, the cross-fertilization between robotics and biology could be one of the most fruitful of the next decade.

## Data availability statement

The original contributions presented in the study are included in the article/supplementary material, further inquiries can be directed to the corresponding author.

## Author contributions

RS: Conceptualization, Investigation, Visualization, Writing – original draft, Writing -review & editing.
